# Activation of TWEAK/Fn14 signaling suppresses TRAFs/NF-?B pathway in the pathogenesis of cancer

**DOI:** 10.17179/excli2021-3378

**Published:** 2021-02-04

**Authors:** Gaurav Gupta, Imran Kazmi, Fahad A. Al-Abbasi, Yogendra Singh, S Roshan, Suneha Rani, Anurag Mishra, Parteek Prasher, Niraj Kumar Jha, Lakshmi Thangavelu, Harish Dureja, Sachin K. Singh, Shvetank Bhatt, Dinesh Kumar Chellappan, Kamal Dua

**Affiliations:** 1 School of Pharmacy, Suresh Gyan Vihar University, Jagatpura 302017, Mahal Road, Jaipur, India; 2 Department of Biochemistry, Faculty of Science, King Abdulaziz University, Jeddah, Saudi Arabia; 3 Maharishi Arvind College of Pharmacy, Ambabari Circle, Ambabari, Jaipur, India; 4 Deccan School of Pharmacy, Aghapura, Hyderabad-01, India; 5 School of Pharmaceutical Sciences, Shri Guru Ram Rai University, Patel Nagar, Dehradun, India; 6 Department of Chemistry, University of Petroleum & Energy Studies, Dehradun 248007, India; 7 Department of Biotechnology, School of Engineering & Technology (SET), Sharda University, Plot No. 32-34 Knowledge Park III Greater Noida, Uttar Pradesh, 201310, India; 8 Department of Pharmacology, Saveetha Dental College and Hospitals, Saveetha Institute of Medical and Technical Sciences, Saveetha University, Chennai, India; 9 Faculty of Pharmaceutical Sciences, Maharshi Dayanand University, Rohtak, India; 10 School of Pharmaceutical Sciences, Lovely Professional University, Phagwara, Punjab, 144411, India; 11 Amity Institute of Pharmacy, Amity University Madhya Pradesh (AUMP), Gwalior, 474005, Madhya Pradesh, India; 12 Department of Life Sciences, School of Pharmacy, International Medical University, Bukit Jalil 57000, Kuala Lumpur, Malaysia; 13 Discipline of Pharmacy, Graduate School of Health, University of Technology Sydney, NSW 2007, Australia

## ⁯

***Dear Editor,***

TNF-related weak inducer of apoptosis (TWEAK) belongs to the TNF superfamily, which expresses its biological effects in several tissues. Being a small pleiotropic cytokine, TWEAK is essentially involved in the regulation of cell death involving necrosis or apoptosis, differentiation, angiogenesis, migration, proliferation, and upregulating the expression of key inflammatory cytokines. As a membrane anchored protein, TWEAK acts by binding to the fibroblast growth factor-inducible 14 (Fn14), which is typically a type I transmembrane protein (Bernardi et al., 2019[1]). This is the only reported ligand to which TWEAK binds to. In addition, TWEAK, being a type II transmembrane protein, also clamps itself onto a soluble cytokine by a proprotein convertase enzyme, namely, furin. Therefore, TWEAK binds both its membrane-anchored and soluble isoforms to Fn14. TWEAK is a tripartite glycoprotein, which constitutes an extracellular transmembrane domain, C-terminal region, and an intracellular region N-terminal (Claus et al., 2018[2]). Fn14, thus, carries the extracellular TWEAK region, as well as, the cytoplasmic tail domain which is necessary for signal transduction. TWEAK-Fn14 expression is primarily located in weakened cells and also in certain conditions involving inflammation and cancers, introducing several downstream signaling channels to reshape these tissues (Dwyer et al., 2020[3]).

TWEAK-Fn14 expression is limited in normal tissues, but is highly expressed in other tumorous conditions and generally in cancer cells. There is an overexpression of this TWEAK system in the ovarian, esophageal, liver, bladder, lung, breast, prostate, colorectal and pancreatic cancers. Increased Fn14 expression is also reported in neuroblastoma, brain glioma and melanoma. Furthermore, several studies have also suggested strong display of Fn14 in breast metastases, non-small cell lung, melanoma, colorectal, and prostate cancers (El-Taweel et al., 2020[4]). Fn14 overexpression is correlated primarily with advanced tumor stage and poor clinical findings.

Fn14 works on cells by triggering the binding of several adapter molecules to downstream molecules thereby causing transduction of signals. The TNF receptor associated factors (TRAF) family contains a variety of essential adapter molecules, which of these are connected to Fn14 and downstream signaling paths (El-Taweel et al., 2020[5]). TRAFs 1-5 have been reported to bind to the cytoplasmic Fn14 tail. The anti-apoptotic protein, TRAF2 binding to Fn14 triggers the activation of NF-κB pathway, which in turn induces several cellular responses. The cellular inhibitor of apoptosis protein 1 (cIAP1) sensitizes various cells to TNF-α and turns on the signals of NF-κB. The presence of TWEAK/Fn14 facilitates the lysosomal degradation of cIAP1-TRAF2, and then sensitizes the TNF-α-induced death immortalized tumor cells (Hu et al., 2017[6]). However, this mechanism is resistant to primary cells. The functional mechanisms in these two cell types could differ in various ways, as TNF-α controls the cell cycle by connecting it to one of the 2 receptors; TNFR (1 or 2). TNFR1 may contribute to cell death, whereas, TNFR2 facilitates the division of cells.

NF-κB induces the production of chemokines, cytokines, metalloproteases and adhesion molecules. This constitutes the key inflammatory pathway leading to the growth and progression of cancer. The signaling of TWEAK/Fn14 triggers all the available alternate NF-κB pathways (Leng et al., 2011[7]). The standard NF-κB pathway is defined by the NF-κB receptor phosphorylation and the p50/p65 heterodimers nuclear translocation, whereas, the alternative NF-kB pathway is stimulated by kinase-inducing NF-κB stabilization and the translocation of heterodimers p52/RelB and p100 into the nucleus. Activity of the TWEAK-stimulated glioblastoma cell invasion is responsible for the NF-κB pathway and the NF-κB active kinase. More-over, TWEAK/Fn14 activates non-canonic NF-κB signals, but it does reveal two distinct findings for melanoma and prostate cancer cells, which are cell invasiveness prevention and stimulation (Liu et al., 2019[8]).

Signaling from TWEAK/Fn14 has closely associated multiple biological tumors. Thus, TWEAK and Fn14 may be used as biomarkers for cancer projections and forecasts. Fn14 is exaggerated in primary breast carcinomas and plays a significant role in the prediction of brain metastasis, with strong specificity. One of the critical observations is that mice with breast carcinoma and brain metastasis, when treated, increased longevity and demonstrated a marked decrease in the production of TWEAK and Fn14 in tumors. Interestingly, the protein Fn14 is linked to human tumor glioma (Liu et al., 2017[9]). The expression of Fn14 is also a significant prediction element in peptic, non-small cell lung cancers, carcinoma and hepatocellular disorder. Higher TWEAK serum rates are correlated with a locoregional deficiency in patients with squamous cell cancer in the neck and head regions, and large expression ratio of CD136/TWEAK in cancer is often associated to weak prognosis. Despite their elevated expression and frequent intervention correlated with tumors, TWEAK and Fn14 are also suitable candidates for tumor therapy (Liu et al., 2016[10]).

A variety of agents aiming at TWEAK or Fn14 were developed in the last decade. They achieved anti-tumor impact via three mechanisms; soluble TWEAK neutralization, Fn14 signal-blockade, and explicit Fn14-mediated tumor cell destruction. RG7212, a monoclonal neutralizing antibody (mAb) which targets TWEAK, may block TWEAK/Fn14 signaling and prevents solid tumor development in patients. RG7212 suppressed TWEAK/Fn14 signaling in preclinical studies and showed activation of NF-κB (Martin-Sanchez et al., 2018[11]). Secretion of cytokines in the cultured tumor cells and in murine models also inhibited the development of tumors. The findings from a Phase I study which was published recently, demonstrated that RG7212 has outstanding tolerability and a good pharmacokinetic profile when systemically administered. In colon cancer mice model, P2D10, anti-TWEAK antibody is shown to decrease diarrhoea induced by 5-Fluorouracil without affecting the anti-tumor effect of 5-Fluorouracil. GrB-TWEAK and GrB-Fc-IT4 are the 2 extremely effective systems with pro-apoptotic serine protease GrB. GrB-TWEAK is made up of the human-binding Fn14 domain as the targeting moiety and GrB-Fc-IT4 is made from a humanized single-chain antibody with Fn14 targeting (El-Taweel et al., 2020[4]). They have also shown affinity and cytotoxicity toward Fn14-mediated liver cancer and tumor prevention *in vivo*. Furthermore, they are fully human made and do not induce patient's immune response. Some of the Fn14-specific antibodies include, 18D1, PDL192, and BIIB036. They have proven to decrease tumor growth *in vivo* by different methods, that include, Fn14 blockade, TWEAK-Fn14 engagement inhibition, and cause cellular cytotoxicity based on an antibody (ADCC). PDL192 and BIIB036 have demonstrated remarkable *in vivo* efficacy, that is at least partially mediated by ADCC and due to the amplification of NF-κB signals. Therefore, the NF-κB pathway is stimulated by BIIB036. It also triggers IL-8 production and causes the destruction of human cancerous cells. The 19D2 antibody prevents cancer development of RENCA cells primarily by ADCC (Zhang et al., 2020[13]). The findings of a Phase I clinical trial suggested that, PDL 192 expressed severe liver and pancreatic medication toxicities at therapeutic dosage levels. These findings warrant further detailed clinical research, which is essentially required to evaluate the protection and efficacy of the Fn14-specific monoclonal antibodies (Fn14 mAbs) (Xu et al., 2016[12]).

The signaling system of TWEAK/Fn14 is active in tumor pathogenesis. It plays a significant role in the development of cancer cells, their proliferation and invasion. TWEAK/Fn14 activation often stimulates downstream signals to control several primary events correlated with tumor proliferation, angiogenesis and EMT. Furthermore, Fn14 may also be an indicator for tumor growth.

## Conflict of interest

The authors declare no conflict of interest.
